# Beyond the Scale: The Hidden Burden of Underweight and Cachexia in Adults with Congenital Heart Defects and Heart Failure—Results from the Pathfinder CHD-Registry

**DOI:** 10.3390/jcm14124355

**Published:** 2025-06-18

**Authors:** Ann-Sophie Kaemmerer-Suleiman, Sebastian Freilinger, Annika Freiberger, Oliver Dewald, Stefan Achenbach, Gert Bischoff, Anna Engel, Peter Ewert, Frank Harig, Jürgen Hörer, Stefan Holdenrieder, Harald Kaemmerer, Renate Kaulitz, Frank Klawonn, Detlef Koch, Dirk Mentzner, Nicole Nagdyman, Rhoia Neidenbach, Wolfgang Schmiedeberg, Mathieu N. Suleiman, Elsa Ury, Robert David Pittrow, Leonard Bernhard Pittrow, Benjamin Alexander Pittrow, Fabian von Scheidt, Wolfgang Wagener, Nicole Wolfrum, Michael Huntgeburth, Fritz Mellert

**Affiliations:** 1 Department of Cardiac Surgery, University Hospital Erlangen, Friedrich-Alexander-University Erlangen-Nürnberg, 91054 Erlangen, Germany; oliver.dewald@fau.de (O.D.); frank.harig@uk-erlangen.de (F.H.); mathieu.suleiman@uk-erlangen.de (M.N.S.);; 2 International Center for Adults with Congenital Heart Disease, Department of Congenital Heart Disease and Paediatric Cardiology, German Heart Centre Munich, Technical University of Munich, 80333 Munich, Germanynagdyman@dhm.mhn.de (N.N.);; 3 Department of Cardiology, Friedrich-Alexander-University of Erlangen-Nürnberg, 91054 Erlangen, Germany; 4 Department of Clinical Nutrition and Preventive Medicine, Hospital Barmherzige Brüder, 93049 Munich, Germany; 5 Department of Congenital and Paediatric Heart Surgery, German Heart Center, University Hospital, Technical University of Munich, 80636 Munich, Germany; 6 Division of Congenital and Pediatric Heart Surgery, University Hospital, Ludwg-Maximilians-University Munich, 81377 Munich, Germany; 7 Department of Laboratory Medicine, German Heart Centre Munich, Technical University of Munich, 80333 Munich, Germany; 8 Pädiatrische Kardiologie, Universitätsklinikum Tübingen, 72076 Tübingen, Germany; renate.kaulitz@med.uni-tuebingen.de; 9 Helmholtz Centre for Infection Research, Biostatistics, Technical University Braunschweig, 38106 Braunschweig, Germany; 10 Deutsche Rentenversicherung Rheinland, 40215 Düsseldorf, Germany; detlef.koch@drv-rheinland.de (D.K.);; 11 Department of Sport and Human Movement Sciencve, University of Vienna, 1010 Vienna, Austria; rhoia.neidenbach@gmail.com

**Keywords:** adults congenital heart disease, heart failure, registry, pathfinder, underweight, cachexia

## Abstract

**Background/Objectives:** Heart failure (HF) poses a major challenge in managing adults with congenital heart defects (ACHD). Emerging evidence suggests that HF in ACHD increases the risk of underweight due to heightened metabolic demands, gastrointestinal complications, and psychological factors such as anxiety and depression. Despite its critical implications, few studies have examined this association. This study evaluates the relationship between HF and underweight—defined as a body mass index (BMI) < 18.5—in ACHD. **Methods:** The Pathfinder-CHD Registry is a prospective, observational, web-based HF registry including ACHD with manifest HF, history of HF, or significant risk for HF. It documents congenital diagnoses, HF type, comorbidities, and treatments. Patients were categorized by BMI into mild (17.00–18.49), moderate (16.00–16.99), and severe (<16.00) underweight. **Results:** As of September 2024, the registry enrolled 1420 adults (mean age 31.8±11.3 years; 49.2% female). Underweight was present in 59 patients (4.2%): 62.7% mild, 18.6% moderate, and 18.6% severe. Among the remaining 1361 patients, 52.8% had normal weight, 32.8% were overweight, and 14.2% were obese. Women had significantly lower metabolic body weight than men (*p* = 0.002). Underweight correlated with younger age (*p* < 0.001) and CHD type (*p* = 0.02). Notably, 42.9% of underweight patients had cyanotic CHD. **Conclusions:** Underweight is an underrecognized problem in ACHD with HF. Adults with complex CHD or connective tissue disorders are disproportionately affected. Underweight should be seen as an alarm sign requiring personalized, multidisciplinary management, including nutritional support, tailored therapy, and close monitoring to improve outcomes.

## 1. Introduction

Heart failure (HF) is one of the most severe long-term problems in the clinical course of adults with congenital heart disease (ACHD), accounting for 25% to 50% of deaths [1,2]. A serious, multifactorial complication of HF is cardiac cachexia, a severe form of muscle and fat wasting that is not only the result of inadequate caloric intake but also of a complex metabolic syndrome driven by systemic inflammation, altered energy metabolism, and neurohormonal changes [3]. In advanced HF, systemic inflammation is accompanied by elevated levels of pro-inflammatory cytokines, such as tumor necrosis factor-alpha (TNF-α), interleukin-1 (IL-1), and interleukin-6 (IL-6), which induce muscle proteolysis through the ubiquitin-proteasome pathway [4]. Chronic systemic inflammation also impairs appetite regulation and contributes to anorexia, further exacerbating the catabolic state. Furthermore, the neurohormonal system, including the renin–angiotensin–aldosterone system (RAAS) and the sympathetic nervous system (SNS), is chronically activated to maintain cardiac output [5]. However, a prolonged activation of these systems can have detrimental effects on skeletal muscle and metabolic function. Elevated angiotensin II and norepinephrine levels promote muscle wasting by increasing oxidative stress, impairing mitochondrial function, and inducing muscle cell apoptosis. In addition, affected patients with HF may exhibit a hypermetabolic state with impaired substrate utilization, leading to the characteristic muscle wasting seen in cachexia [6]. Accompanying gastrointestinal complications, such as intestinal or liver congestion, intestinal edema, and impaired perfusion, can lead to malabsorption of essential nutrients [4]. Additionally, decreased appetite may contribute to reduced food intake, further worsening nutritional deficiencies.

Despite its clinical importance, cachexia in ACHD with HF remains underrecognized and underappreciated in both research and clinical practice [7]. Accordingly, the aim of our study was to identify and evaluate underweight and cachexia in adults with native CHD, after surgical cardiac repair, or after interventional treatment to further the understanding of this rare but very serious complication.

## 2. Materials and Methods

### 2.1. The Prospective Pathfinder-ACHD Registry

The prospective Pathfinder-CHD Registry is a prospective, observational, web-based heart failure registry, established in 2022 in Germany [8].

This clinical study represents a collaborative effort between the Clinic for Congenital Heart Disease and Pediatric Cardiology at the German Heart Center Munich, Technical University Munich, Munich, Germany, the University Hospital Tübingen, Pediatric Cardiology, Tübingen, Germany and the Department for Cardiac Surgery at Friedrich-Alexander-University Erlangen-Nürnberg, Erlangen, Germany.

### 2.2. Study Cohort

Included were ACHD with any form of manifest HF, history of HF, or significant risk for future HF due to abnormal ventricular function or anatomy.

In our study, the phrase “significant risk for heart failure (HF)” refers to ACHD who, despite not currently meeting clinical criteria for manifest HF, present with structural or functional abnormalities known to predispose them to HF. This particularly includes patients with systemic ventricular dysfunction (even if asymptomatic), significant residual lesions (e.g., valvular regurgitation or obstruction), severe pulmonary hypertension, chronically elevated central venous pressure, or a history of arrhythmias, protein-losing enteropathy, or Fontan circulation.

While no single risk score was applied, inclusion was guided by the consensus of experienced ACHD specialists as outlined in the Pathfinder-CHD registry protocol [8].

The registry provides comprehensive documentation of the underlying CHD, type of HF, as well as medical, surgical, and/or interventional treatments, and comorbidities.

Inclusion criteria for the current analysis were as follows: Adults (≥18 years) with a confirmed diagnosis of CHD—either manifest HF (according to ESC criteria), a documented history of HF, or considered at high risk due to systemic ventricular dysfunction, residual lesions, severe pulmonary hypertension, Fontan physiology, or other clinically relevant indicators of HF vulnerability.

Exclusion criteria were the inability to provide informed consent and cognitive impairment that prevented participation in study procedures.

Patients were consecutively enrolled during routine visits or hospitalizations at participating tertiary ACHD centers. To avoid bias, no preselection was applied beyond the criteria above. Data collection was performed using a standardized electronic case report form and included demographic information, cardiac history, medications, anthropometric parameters (BMI, metabolic body weight), functional class, surgical/interventional history, and comorbidities.

Based on the BMI, the study population was categorized into mild (BMI 17.00–18.49), moderate (BMI 16.00–16.99), and severe underweight (BMI < 16.00). However, BMI fails to take account of the body’s composition of fat, water, muscle, and bone, or where the fat is distributed. While MBW was primarily used in animal physiology, it is increasingly applied in human medicine to provide more precise insights into energy requirements, especially in critical clinical conditions. Therefore, in addition, the metabolic body weight (MBW), which describes the relationship between body weight and metabolic rate was calculated [9,10,11].

### 2.3. Ethics Approval and Consent to Participate

The survey has been approved by the institutional ethics review boards of the Technical University Munich (Reference Nr: 158/19S) and of the Friedrich-Alexander-University Erlangen-Nürnberg (Reference Nr.:22-56-Bn). Written informed consent was obtained from all patients before the start of documentation. Guidelines on good pharmacoepidemiological practice (GPP) and data protection guidelines were followed. (Figure S1: Flowchart of Patient Inclusion and Exclusion criteria in the PATHFINDER-CHD Registry and Current Substudy).

### 2.4. Statistical Analysis

The data analysis was performed using SPSS 28.0 (IBM Inc., Armonk, NY, USA). All statistical evaluations of the data were pseudonymized and not person related.

Descriptive statistical methods were used for data analysis and initial characterization of the study population. Differences between the groups were checked and evaluated using ANOVA, *t*-, respectively, one sided χ^2^-tests. Continuous data was expressed as mean ± standard deviation, categorical, or interval scaled variables as absolute numbers or percentages. All occurring *p*-values and tests for significance were performed two-sided. A *p*-value < 0.05 was considered significant.

## 3. Results

### 3.1. Study Sample, Demographic Characteristics, and Treatment Status of the Underweight Patients

As of September 2024, the registry enrolled n = 1420 ACHD diagnosed with any form of HF as defined above. Of these, a total of n = 59 patients (4.2%) were underweight, with 37 (62.7%) classified as mild, 11 (18.6%) moderate, and 11 (18.6%) as severe (Table 1). Among the other 1361 patients, 718 (52.8%) had normal weight, 446 (32.8%) were overweight and 193 (14.2%) obese.

The mean age of the entire cohort at the time of evaluation was 31.8 ± 11.3 years, ranging from 20 to 67 years. The correlation between age and the level of underweight is presented in Table 2.

In the underweight group, the mean height was 173 ± 13.5 cm (range: 139–200 cm), and the mean body weight was 51.4 ± 8.6 kg (range: 35–66 kg), resulting in a body mass index (BMI) of 17.1 ± 1.1 kg/m^2^ (range: 14.2–18.5 kg/m^2^). Men were significantly taller and heavier than women. Accordingly, the metabolic body weight was significantly lower in women (18.2 ± 2.3 (range: 14.4–22.4)) than in men (20.1 ± 2.3 (range: 15.0–24.2)) (*p* = 0.002) (Table 1).

Among the underweight patients, no significant gender difference was observed, with 30 (50.8%) being male.

The highest prevalence of underweight was observed in 17 patients with complex CHD in the form of conotruncal anomalies (including truncus arteriosus, tetralogy of Fallot, pulmonary with atresia-ventricular septal defect, double outlet right ventricle or transposition of the great arteries), in 16 patients with Marfan syndrome, and in 5 with Ebstein’s anomaly. In 25 cases (43.4%), an underlying primarily CHD was present.

The correlation between the underlying CHD and the severity of the underweight is shown in Table 3.

### 3.2. Functional Status

According to the Munich Heart Failure Classification for ACHD, 35 patients (59.3%) were in class C or D. Despite the complexity of the CHD, 49 (83.1%) were in a favorable functional class I or II according to Perloff (Table 1).

### 3.3. Operative, Interventional and Medical Treatment Status

Of these, 2 (3.4%) were native, 41 (69.5%) primarily postoperative, and 16 (27.2%) primarily postinterventional (Table 4).

### 3.4. Pharmacotherapeutic Treatment Status

At the time of the study, 38 (64.4%) out of the 59 patients were receiving targeted HF-medication, including substances for lowering the systemic resistance or blood pressure (Table 4).

### 3.5. Demographic Characteristics and Treatment Status of the Underweight Patients

A significant correlation was found between underweight status and younger age (*p* < 0.001), and between underweight and type of CHD (*p* = 0.02).

Notably, 42.9% (n = 27) of underweight patients had a primary cyanotic CHD.

## 4. Discussion

Cardiac cachexia represents a critical and often overlooked complication inACHD and HF. This study underscores its profound clinical implications and highlights the need for early recognition and intervention.

To the best of our knowledge, this is the first large-scale study to address the presence of underweight and cachexia in a large cohort of ACHD and HF. In this increasingly rising population, this complication carries profound clinical implications.

In patients with acquired cardiac diseases, cachexia and underweight are not merely symptoms of end-stage HF but a distinct clinical syndrome. Underweight and cachexia, and not only obesity and metabolic syndrome, may aggravate cardiac dysfunction and accelerate the progression of HF [12,13]. Its development is driven by a combination of systemic inflammation, neurohormonal dysregulation, altered metabolism, and nutritional deficiencies, all of which interact to create a vicious cycle of muscle wasting, frailty, and cardiac deterioration.

It is well documented that cachexia significantly worsens the prognosis in the affected patients, increasing mortality risk and severely reducing quality of life. This is largely due to the loss of skeletal muscle mass, which affects pulmonary ventilation and oxygenation by impairing respiratory muscle function, physical strength, and mobility, leading to increased frailty and susceptibility to infections. There may be some parallels here to the sarcopenia seen in older patients. These issues are especially relevant in ACHD, who often have both cardiac and pulmonary abnormalities that can be exacerbated by cachexia [14].

The prevalence of underweight and cachexia in the current study cohort of ACHD, particularly in individuals with complex congenital heart anomalies or syndromic conditions such as Marfan syndrome, may also reflect the intricate interplay between systemic inflammation, metabolic dysregulation, and the unique pathophysiological challenges inherent to ACHD.

In addition, patients with cachexia appear to be prone to mental health comorbidities such as depression, anxiety, and social isolation due to physical deterioration and altered body image. These psychological stressors are particularly pronounced in ACHD, who often face lifelong medical challenges and social difficulties related to their congenital heart conditions [15]. Addressing these psychosocial dimensions through comprehensive, multidisciplinary care may be essential for improving overall outcomes for ACHD as well.

## 5. Challenges in Diagnosing Cachexia in ACHD

One of the major challenges in the management of cachexia in HF, especially in ACHD, is underdiagnosis. Cachexia is often overlooked in clinical practice, partly due to the lack of standardized diagnostic criteria and its often insidious onset.

Current general diagnostic criteria for cardiac cachexia include unintentional weight loss of more than 5% over a 12-month period, alongside symptoms such as fatigue, anorexia, and muscle weakness. These criteria may not capture the early stages of cachexia or account for the individual variability in body composition. However, it is unclear whether these parameters are appropriate for the ACHD population, which often has complex cardiopulmonary interactions and atypical clinical courses, necessitating a more tailored cachexia assessment.

As underweight is a widely under-recognized complication in ACHD with HF, the Pathfinder Registry provides crucial real-world data on the relationship between weight status and the medical, surgical, and/or interventional treatment of the underlying cardiac abnormality in this population. This appears to be particularly true for patients with complex CHD or those with connective tissue diseases, who are particularly affected.

In addition to the fact that the metabolic body weight (MBW) was significantly lower in women, there was interestingly no correlation between underweight and sex in the present study. However, unlike traditional metrics such as BMI, MBW may serve as a valuable metric for evaluating energy requirements and metabolic stress in this population, providing a more nuanced understanding of the metabolic demands for guiding nutritional and therapeutic strategies associated with varying body compositions. The lower MBW in women may indicate increased susceptibility to the catabolic effects of HF, necessitating sex-specific approaches to nutritional support.

## 6. Management of Cachexia

The management of cachexia in HF patients, particularly those with ACHD, remains a formidable challenge.

Nutritional interventions, including high-calorie, high-protein diets, may help to some extent, but their effectiveness is limited by the underlying catabolic state, which may be more pronounced in ACHD due to their complex metabolic abnormalities and lifelong disease burden.

It is largely unknown whether SGLT2-I, RAAS inhibitors, mineralocorticoid receptor antagonists (MRA’s), beta-blockers, digitalis glycosides, and anti-inflammatory agents, which are the cornerstones of regular HF treatment, also address the metabolic abnormalities that drive cachexia. This issue is also important in ACHD, who often have a unique HF pathophysiology compared to those without CHD.

Emerging therapies aimed at modulating inflammation, anabolic pathways, and energy metabolism offer hope for improving outcomes in cachectic HF patients, including ACHD. For example, selective androgen receptor modulators (SARMs) and ghrelin mimetics, an endogenous appetite-stimulating peptide hormone, have shown promise in promoting muscle growth and appetite stimulation [16].

However, given that ACHD often undergo multiple medication regimens, the integration of these new therapies into existing treatment plans remains uncertain. In addition, ACHD often require multidisciplinary care, where individualized treatment strategies, including those addressing cachexia, are critical.

As many patients included in our study often come from far away, structured nutritional interventions or anabolic therapies were not systematically implemented, indicating a current gap in care. However, the referring physician was kept informed, and in special cases, patients were admitted to our institution or referred to a cooperating specialist in nutritional medicine.

Chronic inflammation was not systematically quantified in the studied cohort. Many patients seen in the outpatient cardiology clinics were referred with existing laboratory results provided by their general practitioners. To avoid redundant diagnostics and unnecessary blood draws, specific recommendations were made for referring physicians to include inflammatory markers, such as CRP, erythrocyte sedimentation rate, IL-6, and complete blood count, during routine follow-up testing, especially in clinically stable patients.

In more critical or unstable cases, appropriate laboratory diagnostics were performed within the clinical setting. However, these values were collected solely for clinical management purposes and were not analyzed systematically for research.

## 7. Study Limitations

The strengths of this study include the large sample size of ACHD with HF for this condition. However, limitations must be considered when interpreting the current results.

As the classification was based on BMI, the underweight patients certainly had significant malnutrition. To better reflect disease-related malnutrition, it would be advisable for future studies to diagnose malnutrition according to the GLIM criteria (Global Leadership Initiative on Malnutrition) [17]. This would also make it easier to distinguish between normal weight malnourished patients and obese malnourished patients (‘sarcopenic obesity’).

A primary limitation of this study is the relatively small sample size of underweight patients (n = 59) analyzed from a larger registry cohort, which may limit the statistical power and generalizability of the findings.

Furthermore, the sample of HF patients treated in tertiary care centers does not represent the typical population of these patients treated by general practitioners, internists, or general cardiologists. The prevalence of severe forms of ACHD with HF in a specialized tertiary care center is likely to be higher than in general care hospitals or even cardiology departments. Furthermore, the data presented are exclusively from patients living in Germany. Generalizing the conclusions and applying them to patients living in other countries or cultural contexts is questionable.

## 8. Conclusions

Underweight and cachexia are underrecognized, but clinically relevant, complications in ACHD, particularly in combination with heart failure. Our findings highlight the need to view underweight status as a critical warning sign, particularly in patients with complex CHD or syndromic conditions.

Early identification and a multidisciplinary approach, combining nutritional support, tailored HF therapy, and close monitoring, are essential to improving outcomes.

Although current treatment options are limited, emerging therapies offer promise for addressing the underlying metabolic disturbances.

Future research should focus on early intervention strategies and long-term outcomes in this vulnerable population. Moreover, early detection of this problem can help to initiate appropriate health promotion, prevention, and rehabilitation measures. This can be achieved not only by specialist centers for ACHD, but also and especially in the primary care system.

## Figures and Tables

**Table 1:** Demographic parameters, overall and split by sex.

Demographics	Overall (N = 59)	Women (n = 30)	Men (n = 29)	*p*-Value
**Age [years]**	31.8 ± 11.3 (range: 20–67)	33.6 ± 10.0 (range: 20–61)	29.9 ± 12.5 (range: 20–67)	0.208
**Weight [kg]**	51.4 ± 8.6 (range: 35–66)	48.1 ± 7.8 (range: 35–63)	54.8 ± 8.1 (range: 37–66)	**0.002** *
**Metabolic Body Weight [kg]**	19.1 ± 2.5 (range: 14.4–24.2)	18.2 ± 2.3 (range: 14.4–22.4)	20.1 ± 2.3 (range: 15.0–24.2)	**0.002** *
**Height [m]**	173.0 ± 13.5 (range: 139–200)	167.2 ± 13.1 (range: 139–191)	179.1 ± 11.2 (range: 157–200)	**<0.001** *
**Dubois Body Surface Area [m^2^]**	1.61 ± 0.20 (range: 1.16–1.99)	1.52 ± 0.19 (range: 1.16–1.88)	1.69 ± 0.18 (range: 1.30–1.99)	**<0.001** *
**BMI [kg/m^2^]**	17.1 ± 1.1 (range: 14.2–18.5)	17.1 ± 1.1 (range: 14.2–18.5)	17.0 ± 1.2 (range: 14.7–18.5)	0.613
*Mild Underweight* *(BMI 17–18.5)*	37 (62.7%)	22 (73.3%)	15 (51.7%)	—
*Moderate Underweight (BMI 16–16.9)*	11 (18.6%)	3 (10.0%)	8 (27.6%)	—
*Severe Underweight* *(BMI < 16)*	11 (18.6%)	5 (16.7%)	6 (20.7%)	—
**Functional Class (Perloff)**				**0.024** *
*I/II*	49 (83.1%)	21 (70.0%)	28 (96.6%)	—
*III/IV*	10 (17.0%)	9 (30.0%)	1 (3.4%)	—
**MUC-HF Classification**				0.531
*B*	24 (40.7%)	13 (43.3%)	11 (37.9%)	—
*C*	34 (57.6%)	16 (53.3%)	18 (62.1%)	—
*D*	1 (1.7%)	1 (3.3%)	0 (0.0%)	—
**Previous Cardiac Surgery**	42 (71.2%)	24 (82.8%)	18 (60.0%)	**0.050** *
**Medication Use**	39 (66.1%)	18 (60.0%)	21 (72.4%)	0.314
**Primary Cyanotic CHD**	25 (42.4%)	11 (36.7%)	14 (48.3%)	0.367

*, statistically significant (*p* < 0.05); kg, kilograms; m, meters; BMI, body-mass-index; MUC-HF, Munich Heart Failure.

**Table 2:** BMI-Classification by age group.

Age Group	Mild Underweight (BMI 17–18.5 kg/m^2^)	Moderate Underweight (BMI 16–16.9 kg/m^2^)	Severe Underweight (BMI < 16 kg/m^2^)	*p*-Value
**18–24 (n = 18)**	12 (32.4%)	2 (18.2)	4 (36.4%)	0.381
**25–34 (n = 22)**	10 (27.0%)	6 (54.5%)	6 (54.5%)	
**35–44 (n = 13)**	10 (27.0%)	2 (18.2%)	1 (9.1%)	
**45–54 (n = 5)**	4 (10.8%)	1 (9.1%)	-	-
**55+ (n = 1)**	1 (2.7%)	-	-	-
	37 (62.7%)	11 (18.6%)	11 (18.6%)	

kg, kilograms; m, meters BMI, body-mass-index; n, absolute number.

**Table 3:** The association between underlying congenital heart defect and the extent of underweight.

Age Group	Mild Underweight (BMI 17–18.5 kg/m^2^)	Moderate Underweight (BMI 16–16.9 kg/m^2^)	Severe Underweight (BMI < 16 kg/m^2^)	Metabolic Body Weight
**Aortic valve disease (n = 2)**	2 (100%)	-	-	43.5 ± 4.2
**ccTGA (n = 2)**	-	2 (100%)	-	41.3 ± 5.3
**DILV (n = 3)**	2 (66.6%)	1 (33.3%)	-	37.5 ± 1.5
**Ebstein (n = 5)**	4 (80%)	-	1 (20%)	37.8 ± 5.2
**Eisenmenger (n = 3)**	2 (66.6%)	-	1 (33.3%)	32.8 ± 6.1
**HLHS (n = 2)**	2 (100%)	-	-	45.2 ± 3.0
**Marfan Syndrome (n = 16)**	8 (50%)	4 (25%)	4 (25%)	43.2 ± 5.6
**PA intact VS (n = 1)**	1 (100%)	-	-	27.8
**PA-VSD (n = 5)**	3 (60%)	1 (20%)	1 (20%)	30.2 ± 3.6
**PuR (n = 1)**	-	1 (100%)	-	34.5
**Shone complex (n = 1)**	1 (100%)	-	-	37.5
**TGA (n = 3)**	1 (33.3%)	1 (33.3%)	1 (33.3%)	39.5 ± 4.8
**TOF (n = 9)**	6 (66.6%)	1 (11.1%)	2 (22.2%)	36.8 ± 7.6
**TrA (n = 1)**	1 (100%)	-	-	31.5
**UVH (n = 2)**	2 (100%)	-	-	40.1 ± 2.7
**VSD (n = 3)**	2 (66.6%)	-	1 (33.3%)	35.5 ± 8.3

kg, kilograms; m, meters BMI, body-mass-index; n, absolute number; ccTGA, congenitally corrected transposition of the great arteries; DILV, double inlet left ventricle; Ebstein, Ebstein’s anomaly; Eisenmenger, Eisenmenger syndrome; HLHS, hypoplastic left heart syndrome; Marfan Syndrome, Marfan syndrome; PA-intact VS, pulmonary atresia with intact ventricular septum; PA-VSD, pulmonary atresia with ventricular septal defect; PuR, pulmonary regurgitation; TGA, transposition of the great arteries; TOF, tetralogy of Fallot; TrA, truncus arteriosus communis; UVH, univentricular heart; VSD, ventricular septal defect.

**Table 4:** Clinical characteristics of the included patients.

ID	Age Group	Sex	BMI	Met. BW	Type of CHD	Native/ Post OP/ Post Intervention	Cardiac Medication
001-0020	35–44	female	18.5	17.37	PA-VSD	Post-OP	Beta blockers ACE-I Aldosterone antagonist Oral Anticoagulant
001-0061	25–34	male	15.2	16.79	PA-VSD	Post-OP	ACE-I Aldosterone antagonist Antiarrhytmica Oral Anticoagulant
001-0114	18–24	male	17.8	19.36	DILV	Post-OP	Beta blockers ATB Oral Anticoagulant
001-0126	25–34	female	17.1	17.37	Eisenmenger	Native	PDE5-I
001-0149	35–44	male	18.0	19.92	Aortic valve disease	Post-OP	Beta blockers ATB
001-0217	25–34	female	16.3	18.80	ccTGA	Post-OP	Beta blockers ACE-I Aldosterone antagonist Oral Anticoagulant
001-0392	25–34	male	18.4	18.80	SingleVentricle	Post-OP	Beta blockers ACE-I Oral Anticoagulant ATB
001-0397	18–24	male	18.3	20.85	HLHS	Post-OP	Beta blockers ACE-I Diuretics Oral Anticoagulant Digitalis
001-0405	35–44	female	18.0	17.66	Ebstein	Native	Oral Anticoagulant
001-0453	25–34	female	17.6	17.37	Ebstein	Post-OP	Diuretics Aldosterone antagonist Oral Anticoagulant Iron
001-0470	35–44	male	18.3	22.09	Ebstein	Post-OP	Beta blockers ACE-I Diuretics Digitalis Oral Anticoagulant
001-0501	18–24	female	15.6	14.39	Eisenmenger	Native	PDE5-I Oral Anticoagulant
001-0520	18–24	male	17.1	18.80	Shone-Komplex	Post-OP	Beta blockers ACE-I ATB Oral Anticoagulant
001-0546	25–34	male	16.5	18.24	DILV	Native	none
001-0568	25–34	female	15.2	17.95	Marfan	Post-OP	Beta blockers
001-0571	25–34	male	16.5	24.20	Marfan	Post-OP	PDE5-I
001-0577	25–34	female	17.3	21.56	Marfan	Native	Beta blockers
001-0578	25–34	male	17.6	21.29	Marfan	Post-OP	Beta blockers ATB
001-0583	45–54	male	16.9	22.36	Marfan	Post-OP	ATB
001-0586	35–44	female	17.3	22.36	Marfan	Native	Beta blockers Aldosterone antagonist Antiarrhythmics Oral Anticoagulant
001-0593	25–34	male	16.6	21.56	Marfan	Native	Beta blockers Sacubitril/Valsartan Diuretics Aldosterone antagonist SGLT2-I Oral Anticoagulant PDE5-I ETA
001-0596	35–44	female	18.2	20.74	Marfan	Post-OP	Beta blockers Diuretics Aldosterone antagonist Oral Anticoagulant Iron
001-0599	25–34	male	15.8	18.52	Marfan	Native	Oral Anticoagulant
001-0615	35–44	female	14.2	15.91	Marfan	Native	Oral Anticoagulant
001-0645	45–54	female	17.4	14.39	PA-VSD	Post-OP	none
001-0669	25–34	female	17.3	17.66	PA intact VS	Post-OP	ATB
001-0680	18–24	female	18.1	14.39	PA-VSD	Post-OP	none
001-0703	45–54	female	17.4	16.50	TrA	Post-OP	Beta blockers Diuretics Aldosterone antagonist Oral Anticoagulant Iron
001-0826	25–34	female	17.4	19.08	UVH	Post-OP	Oral Anticoagulant Iron
001-0863	18–24	male	18.5	22.09	Aortic valve disease	Post-OP	none
001-0924	18–24	male	18.5	21.56	TGA	Post-OP	none
001-0968	25–34	male	15.0	15.00	TOF	Post-OP	Oral Anticoagulant PDE5 ETA sGC-stimulator Prostanoid
001-0969	25–34	female	17.6	18.24	TOF	Post-OP	Diuretics Aldosterone antagonist Oral Anticoagulant
001-0980	18–24	male	17.9	22.63	TOF	Post-OP	none
001-0982	18–24	male	14.7	17.08	TOF	Post-OP	Beta blockers
001-0994	45–54	female	17.4	15.00	TOF	Post-OP	Beta blockers Sacubitril/Valsartan Diuretics Aldosterone antagonist
001-1086	35–44	male	16.0	16.50	TOF	Post-OP	Beta blockers Diuretics Aldosterone antagonist SGLT2-I
001-1110	25–34	female	17.5	21.83	Marfan	Native	Beta blockers
001-1113	18–24	male	15.0	21.56	Marfan	Native	Oral Anticoagulant
001-1182	18–24	female	17.9	18.80	TOF	Post-OP	none
001-1212	35–44	female	17.5	16.50	VSD	Post-OP	Beta blockers
001-1260	25–34	female	16.3	17.66	PI	Post-OP	none
001-1263	25–34	male	17.5	21.02	TOF	Post-OP	ATB
001-1283	25–34	female	15.2	15.91	VSD	Post-OP	Beta blockers ETA sGC-Stimulator Prostanoid
001-1287	18–24	male	18.2	22.36	HLHS	Post-OP	Beta blockers ACE-I Oral Anticoagulant
001-1311	18–24	female	17.5	21.56	Marfan	Native	ATB
001-1314	18–24	female	18.0	20.74	Marfan	Native	Oral Anticoagulant
001-1366	18–24	male	16.2	16.79	PA-VSD	Post-OP	Beta blockers
001-1392	18–24	male	17.8	21.83	TOF	Post-OP	none
001-1417	35–44	female	18.1	19.08	Eisenmenger	Native	Oral Anticoagulant PDE5-I sGC-Stimulator Prostanoid
001-1443	25–34	female	15.1	18.24	Ebstein	Post Intervention	Diuretics Aldosterone antagonist
001-1452	35–44	female	18.1	19.08	Ebstein	Post-OP	Diuretics Aldosterone antagonist Oral Anticoagulant
001-1492	25–34	female	16.5	19.08	Marfan	Native	Beta blockers
001-1543	45–54	male	17.3	21.56	VSD	Post-OP	ACE-I Aldosterone antagonist Antiarrhythmics Oral Anticoagulant
001-1548	55–64	male	17.4	22.73	Marfan	Post-OP	Beta blockers Iron
001-1696	35–44	male	16.8	21.56	ccTGA	Native	Diuretics Aldosterone antagonist
004-0001	18–24	male	16.6	18.52	TGA	Post-OP	Beta blocker
004-0015	35–44	female	18.3	20.47	UVH	Post-OP	Beta blocker ETA sGC-stimulator Prostanoid
004-0019	18–24	male	15.8	18.52	TGA	Post Intervention	Oral Anticoagulant PDE5-I

ccTGA, congenitally corrected transposition of the great arteries; DILV, double inlet left ventricle; Ebstein, Ebstein’s anomaly; Eisenmenger, Eisenmenger syndrome; HLHS, hypoplastic left heart syndrome; Marfan, Marfan syndrome; PA-intact VSD, pulmonary atresia with intact ventricular septum; PA-VSD, pulmonary atresia with ventricular septal defect; PI, pulmonary insufficiency; TGA, transposition of the great arteries; TOF, tetralogy of Fallot; TrA, truncus arteriosus communis; UVH, univentricular heart; VSD, ventricular septal defect. Medication: ACE-I; ACE ihibitor; PDE5-I; PDE5-Inhibitor; ETA; Endothelin antagonist; SGLT2-I; SGLT2-Inhibitor

## Data Availability

The original contributions presented in this study are included in the article. Further inquiries can be directed to the corresponding author(s).

## Supplementary Materials

The following supporting information can be downloaded at: https://www.mdpi.com/article/10.3390/jcm14124355/s1, Figure S1: Flowchart of Patient Inclusion and Exclusion criteria in the PATHFINDER-CHD Registry and Current Substudy.

## Abbreviations

The following abbreviations are used in this manuscript:

ACHD	Adults with Congenital Heart Disease
ATB	Antithrombotic Therapy
BMI	Body Mass Index
CHD	Congenital Heart Disease
DILV	Double Inlet Left Ventricle
ETA	Endothelin Antagonist
Ebstein	Ebstein’s Anomaly
GLIM	Global Leadership Initiative on Malnutrition
GPP	Good Pharmacoepidemiological Practice
HF	Heart Failure
HLHS	Hypoplastic Left Heart Syndrome
IL-1	Interleukin-1
IL-6	Interleukin-6
MBW	Metabolic Body Weight
MRA	Mineralocorticoid Receptor Antagonist
PA-VSD	Pulmonary Atresia with Ventricular Septal Defect
PA-intact VSD	Pulmonary Atresia with Intact Ventricular Septum
PDE5-I	Phosphodiesterase-5 Inhibitor
PI	Pulmonary Insufficiency
PuR	Pulmonary Regurgitation
RAAS	Renin–Angiotensin–Aldosterone System
SARMs	Selective Androgen Receptor Modulators
SGLT2-I	Sodium-Glucose Cotransporter 2 Inhibitor
SNS	Sympathetic Nervous System
SPSS	Statistical Package for the Social Sciences
TGA	Transposition of the Great Arteries
TNF-α	Tumor Necrosis Factor Alpha
TOF	Tetralogy of Fallot
TrA	Truncus Arteriosus Communis
UVH	Univentricular Heart
VSD	Ventricular Septal Defect
ccTGA	Congenitally Corrected Transposition of the Great Arteries
sGC-stimulator	Soluble Guanylate Cyclase Stimulator

## References

[1] Kaemmerer H., Freilinger S., Neidenbach R., Achenbach S., Andonian C., Ewert P., de Haan F., Nagdyman N., Schelling J., Hofbeck M. (2022). Care of adults with congenital heart diseases in Germany-Leading role by internal medicine specialists and general practitioners. Internist.

[2] Ladouceur M., Bouchardy J. (2024). Epidemiology and Definition of Heart Failure in Adult Congenital Heart Disease. Heart Fail. Clin..

[3] Thanapholsart J., Khan E., Ismail T.F., Lee G.A. (2023). The complex pathophysiology of cardiac cachexia: A review of current pathophysiology and implications for clinical practice. Am. J. Med. Sci..

[4] Soto M.E., Perez-Torres I., Rubio-Ruiz M.E., Manzano-Pech L., Guarner-Lans V. (2022). Interconnection between Cardiac Cachexia and Heart Failure-Protective Role of Cardiac Obesity. Cells.

[5] Krysztofiak H., Wleklik M., Migaj J., Dudek M., Uchmanowicz I., Lisiak M., Kubielas G., Straburzyńska-Migaj E., Lesiak M., Kałużna-Oleksy M. (2020). Cardiac Cachexia: A Well-Known but Challenging Complication of Heart Failure. Clin. Interv. Aging.

[6] Lena A., Ebner N., Anker M.S. (2019). Cardiac cachexia. Eur. Heart J. Suppl..

[7] Lena A., Ebner N., Coats A.J.S., Anker M.S. (2019). Cardiac cachexia: The mandate to increase clinician awareness. Curr. Opin. Support. Palliat. Care.

[8] Freilinger S., Kaemmerer H., Pittrow R.D., Achenbach S., Baldus S., Dewald O., Ewert P., Freiberger A., Gorenflo M., Harig F. (2024). PATHFINDER-CHD: Prospective registry on adults with congenital heart disease, abnormal ventricular function, and/or heart failure as a foundation for establishing rehabilitative, prehabilitative, preventive, and health-promoting measures: Rationale, aims, design and methods. BMC Cardiovasc. Disord..

[9] Holliday M.A., Potter D., Jarrah A., Bearg S. (1967). The relation of metabolic rate to body weight and organ size. Pediatr. Res..

[10] Kleiber M. (1947). Body size and metabolic rate. Physiol. Rev..

[11] Kaemmerer K. (1955). Der Energiebedarf landwirtschaftlicher Nutztiere. Z. Für Tierphysiol. Tierernährung Und Futtermittelkunde.

[12] Deen J.F., Krieger E.V., Slee A.E., Arslan A., Arterburn D., Stout K.K., Portman M.A. (2016). Metabolic Syndrome in Adults With Congenital Heart Disease. J. Am. Heart Assoc..

[13] Lembo M., Strisciuglio T., Fonderico C., Mancusi C., Izzo R., Trimarco V., Bellis A., Barbato E., Esposito G., Morisco C. (2024). Obesity: The perfect storm for heart failure. ESC Heart Fail..

[14] Steinmetz C., Krause L., Sulejmanovic S., Kaumkotter S., Mengden T., Grefe C., Knoglinger E., Reiss N., Brixius K., Bjarnason-Wehrens B. (2024). The prevalence and impact of sarcopenia in older cardiac patients undergoing inpatient cardiac rehabilitation—Results from a prospective, observational cohort pre-study. BMC Geriatr..

[15] Freiberger A., Richter C., Huber M., Beckmann J., Freilinger S., Kaemmerer H., Ewert P., Kohls N., Henningsen P., Allwang C. (2023). Post-Traumatic Distress in Adults With Congenital Heart Disease: An Under-Recognized Complication?. Am. J. Cardiol..

[16] Lund L.H., Hage C., Pironti G., Thorvaldsen T., Ljung-Faxen U., Zabarovskaja S., Shahgaldi K., Webb D.-L., Hellström P.M., Andersson D.C. (2023). Acyl ghrelin improves cardiac function in heart failure and increases fractional shortening in cardiomyocytes without calcium mobilization. Eur. Heart J..

[17] Cederholm T., Jensen G.L., Correia M., Gonzalez M.C., Fukushima R., Higashiguchi T., Baptista G., Barazzoni R., Blaauw R., Coats A. (2019). GLIM criteria for the diagnosis of malnutrition—A consensus report from the global clinical nutrition community. J. Cachexia Sarcopenia Muscle.

