# Supplementary figures and images for: Beyond the Scale: The Hidden Burden of Underweight and Cachexia in Adults with Congenital Heart Defects and Heart Failure—Results from the Pathfinder CHD-Registry
Source: J Clin Med. 2025 Jun 18;14(12):4355. doi: 10.3390/jcm14124355 (PMC12194136; doi:10.3390/jcm14124355)

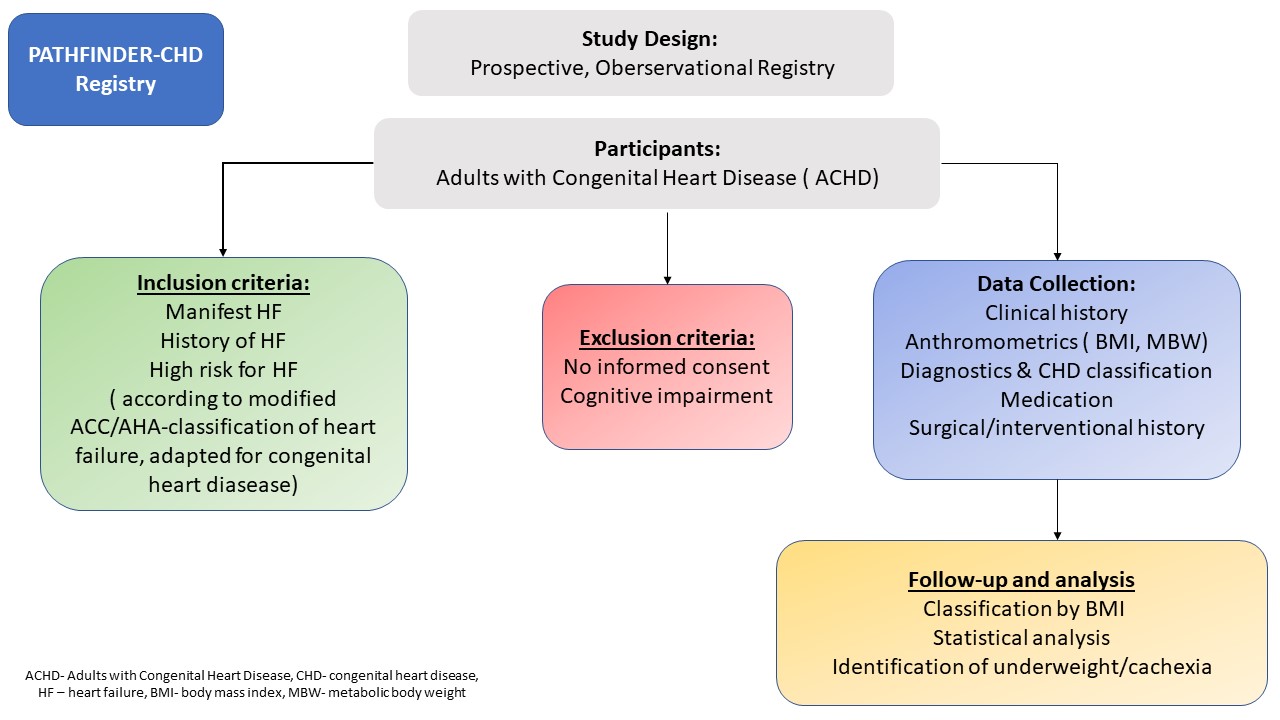

Supplement: Supplementary file 1 [file jcm-14-04355-s001.zip › jcm-3680852-Figure S1.jpg]
